# Supplementary figures and images for: A low-cost wireless extension for object detection and data logging for educational robotics using the ESP-NOW protocol
Source: PeerJ Comput Sci. 2024 Feb 16;10:e1826. doi: 10.7717/peerj-cs.1826 (PMC10909231; doi:10.7717/peerj-cs.1826)

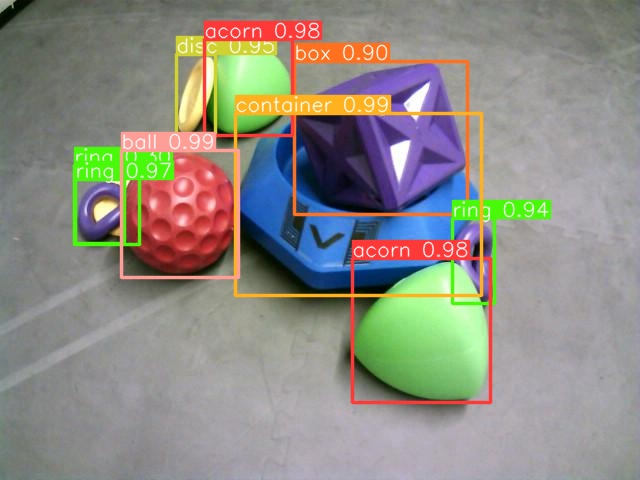

Supplement: Supplemental Information 4 — All of the model parameters can be found in the game.yaml file, the model weights can be found in TrainedModel_V1/weights/best.pt. The validation step has labeled and predicted images in TrainedModel_V1/val_batch0_labels and TrainedModel_V1/val_batch0_pred. [file peerj-cs-10-1826-s004.zip › YoloV8/predict2/8212b7eb-05_18-10_34_21_AM_img.jpg]

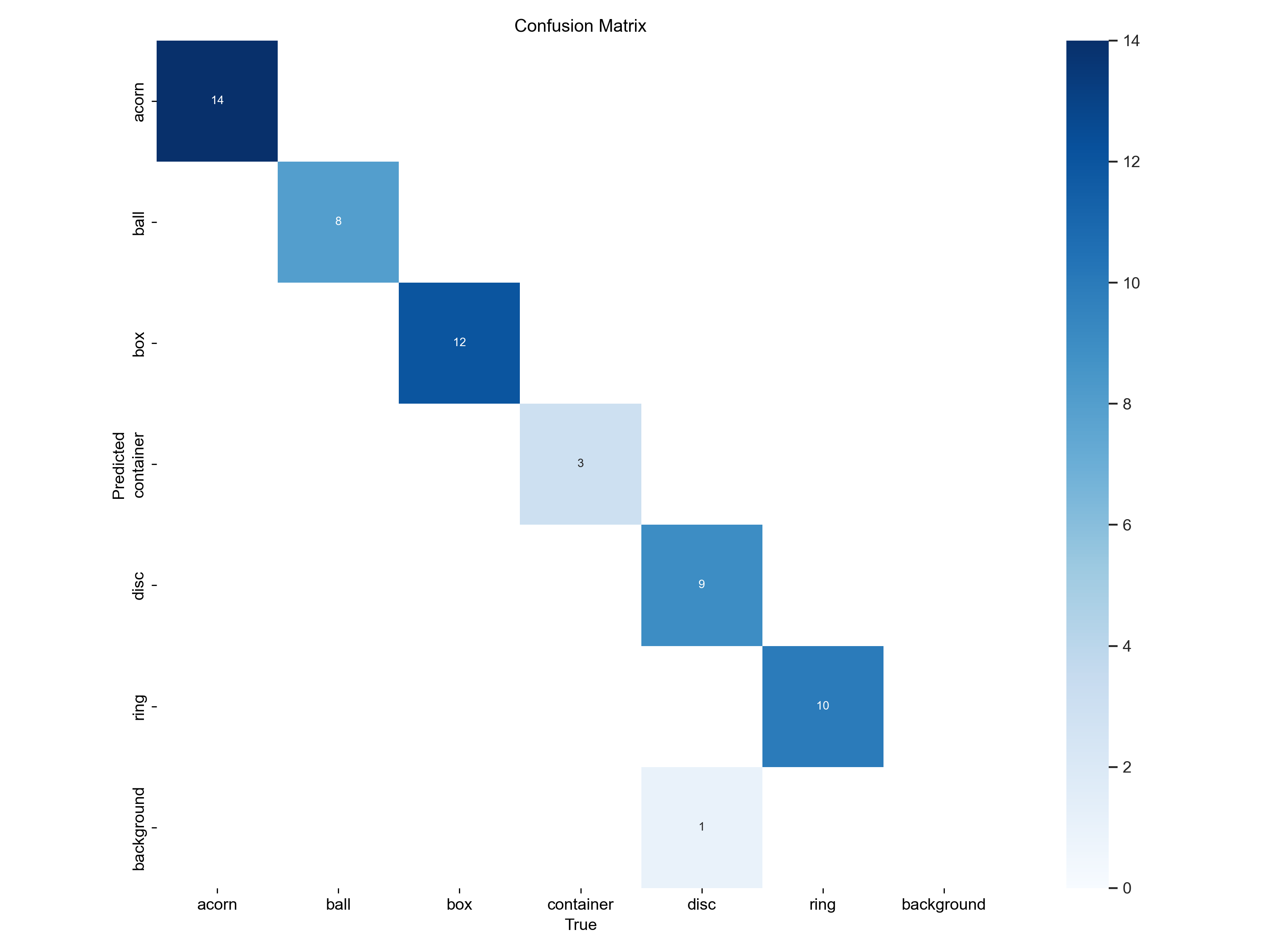

Supplement: Supplemental Information 4 — All of the model parameters can be found in the game.yaml file, the model weights can be found in TrainedModel_V1/weights/best.pt. The validation step has labeled and predicted images in TrainedModel_V1/val_batch0_labels and TrainedModel_V1/val_batch0_pred. [file peerj-cs-10-1826-s004.zip › YoloV8/TrainedModel_V1/confusion_matrix.png]

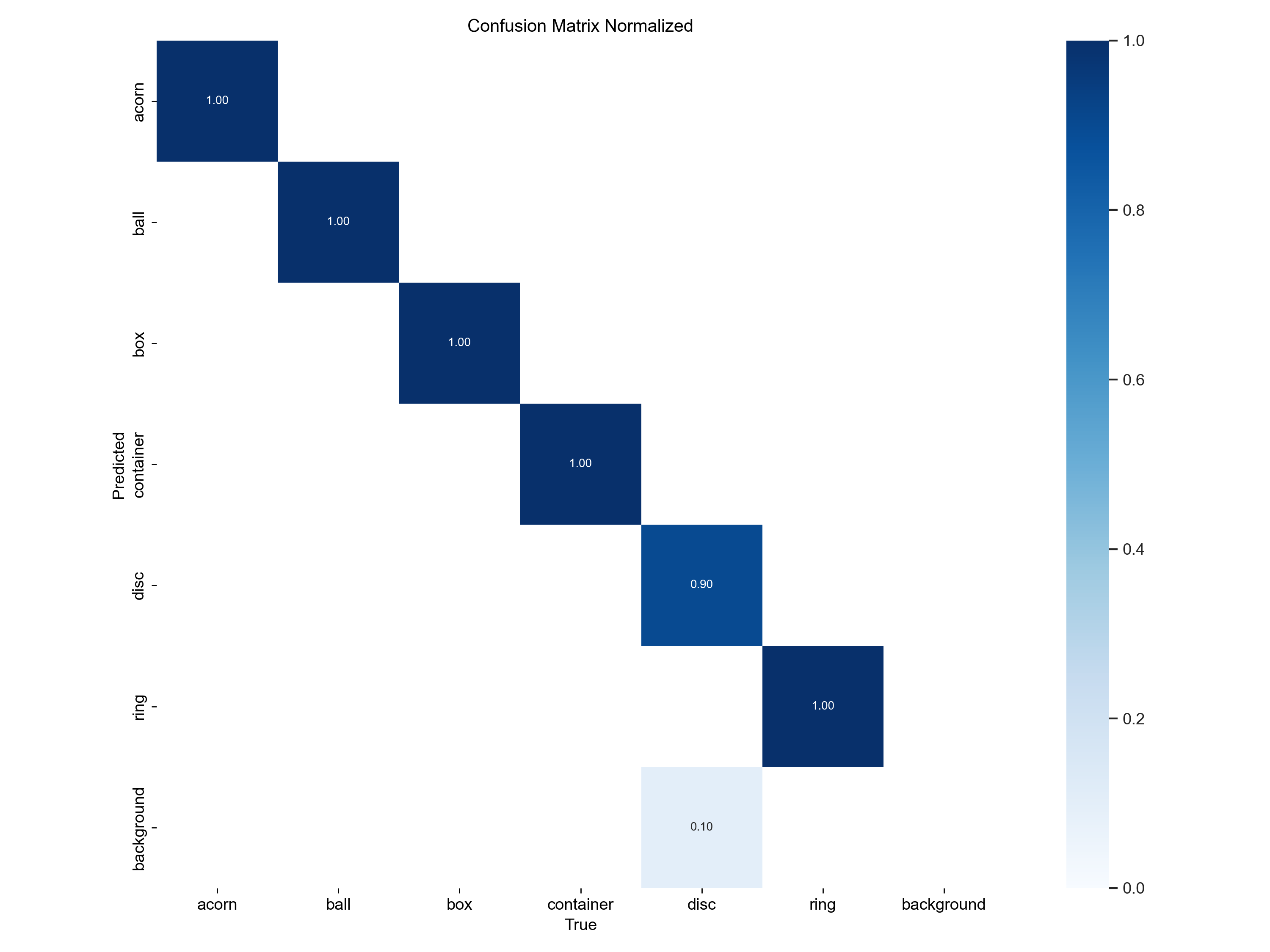

Supplement: Supplemental Information 4 — All of the model parameters can be found in the game.yaml file, the model weights can be found in TrainedModel_V1/weights/best.pt. The validation step has labeled and predicted images in TrainedModel_V1/val_batch0_labels and TrainedModel_V1/val_batch0_pred. [file peerj-cs-10-1826-s004.zip › YoloV8/TrainedModel_V1/confusion_matrix_normalized.png]

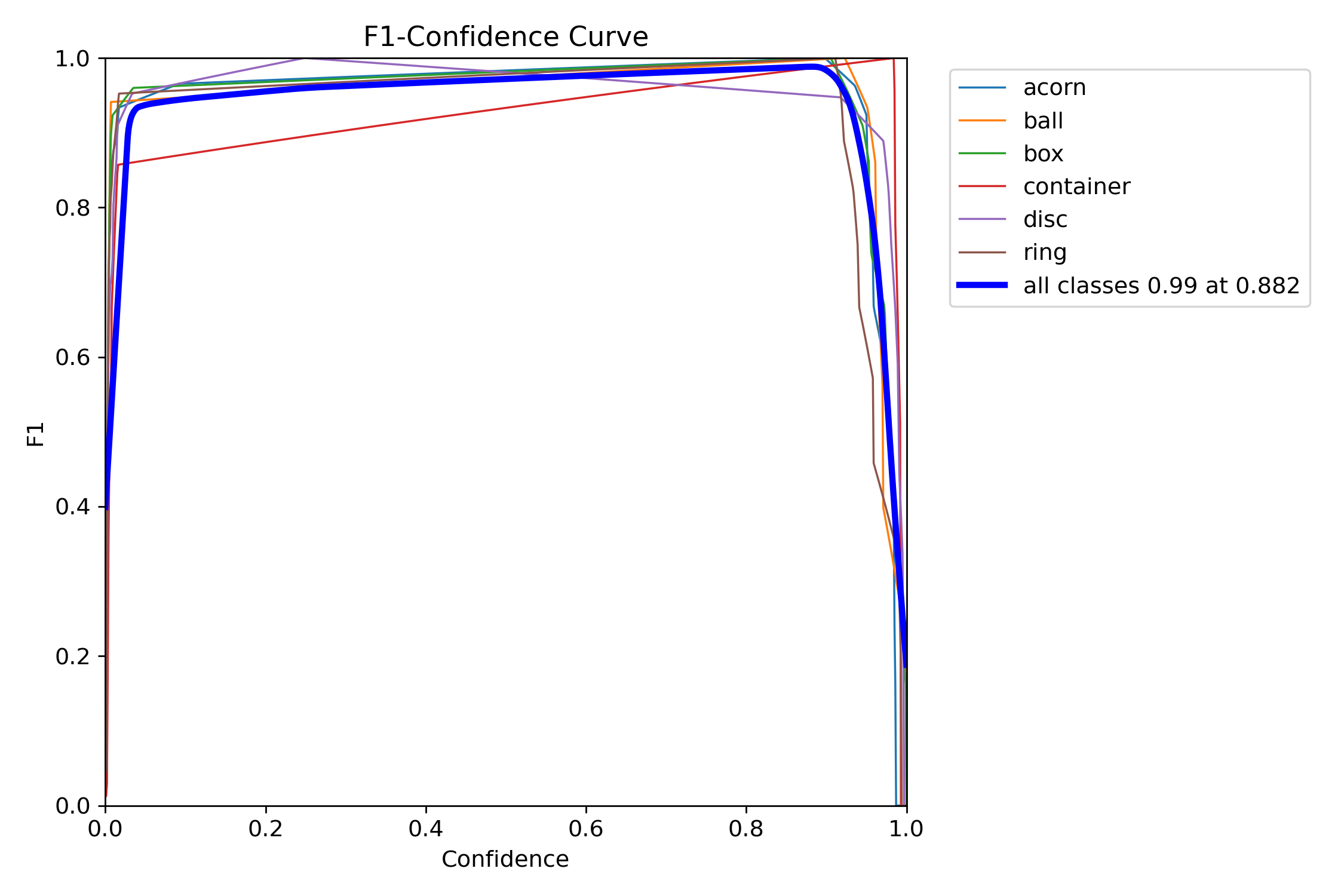

Supplement: Supplemental Information 4 — All of the model parameters can be found in the game.yaml file, the model weights can be found in TrainedModel_V1/weights/best.pt. The validation step has labeled and predicted images in TrainedModel_V1/val_batch0_labels and TrainedModel_V1/val_batch0_pred. [file peerj-cs-10-1826-s004.zip › YoloV8/TrainedModel_V1/F1_curve.png]

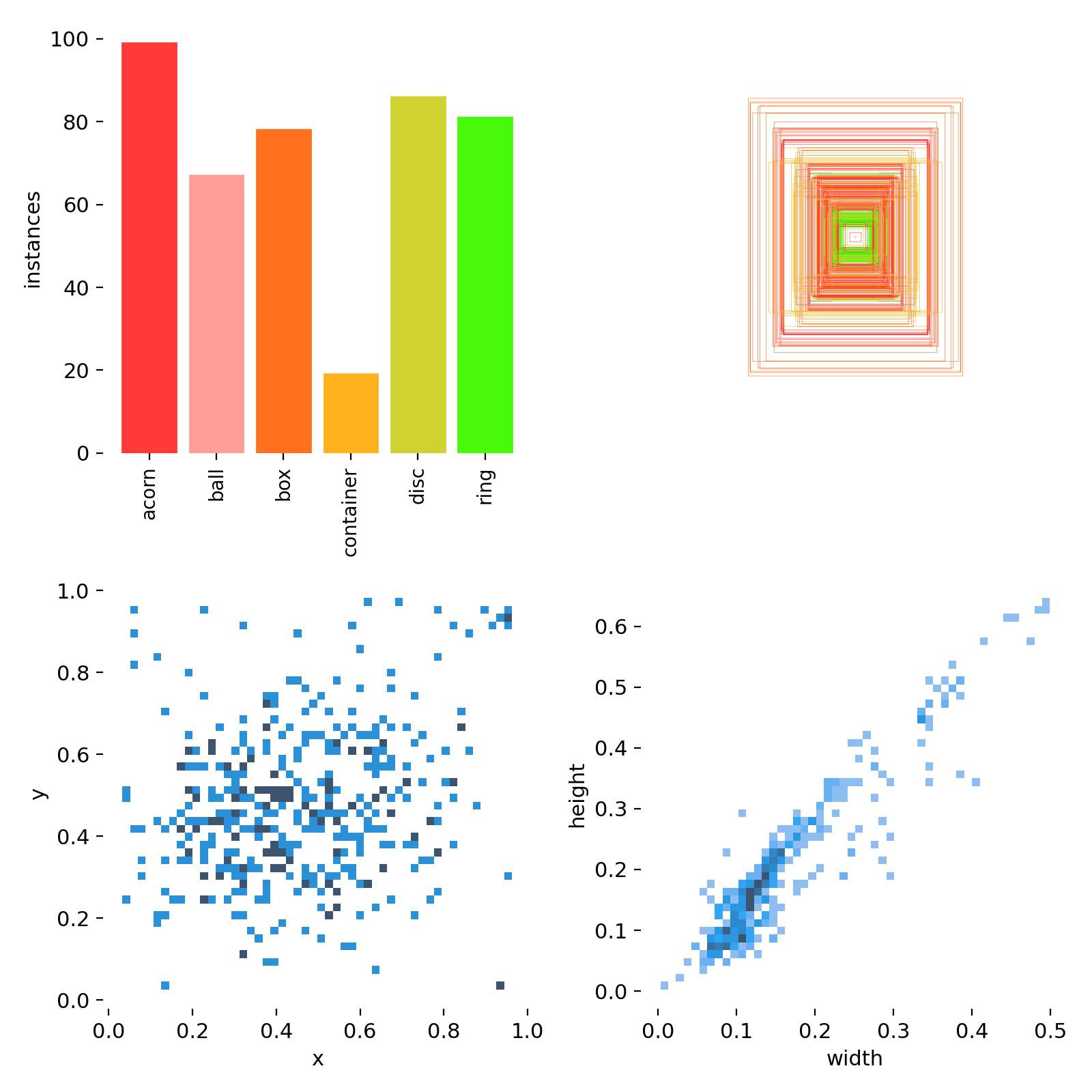

Supplement: Supplemental Information 4 — All of the model parameters can be found in the game.yaml file, the model weights can be found in TrainedModel_V1/weights/best.pt. The validation step has labeled and predicted images in TrainedModel_V1/val_batch0_labels and TrainedModel_V1/val_batch0_pred. [file peerj-cs-10-1826-s004.zip › YoloV8/TrainedModel_V1/labels.jpg]

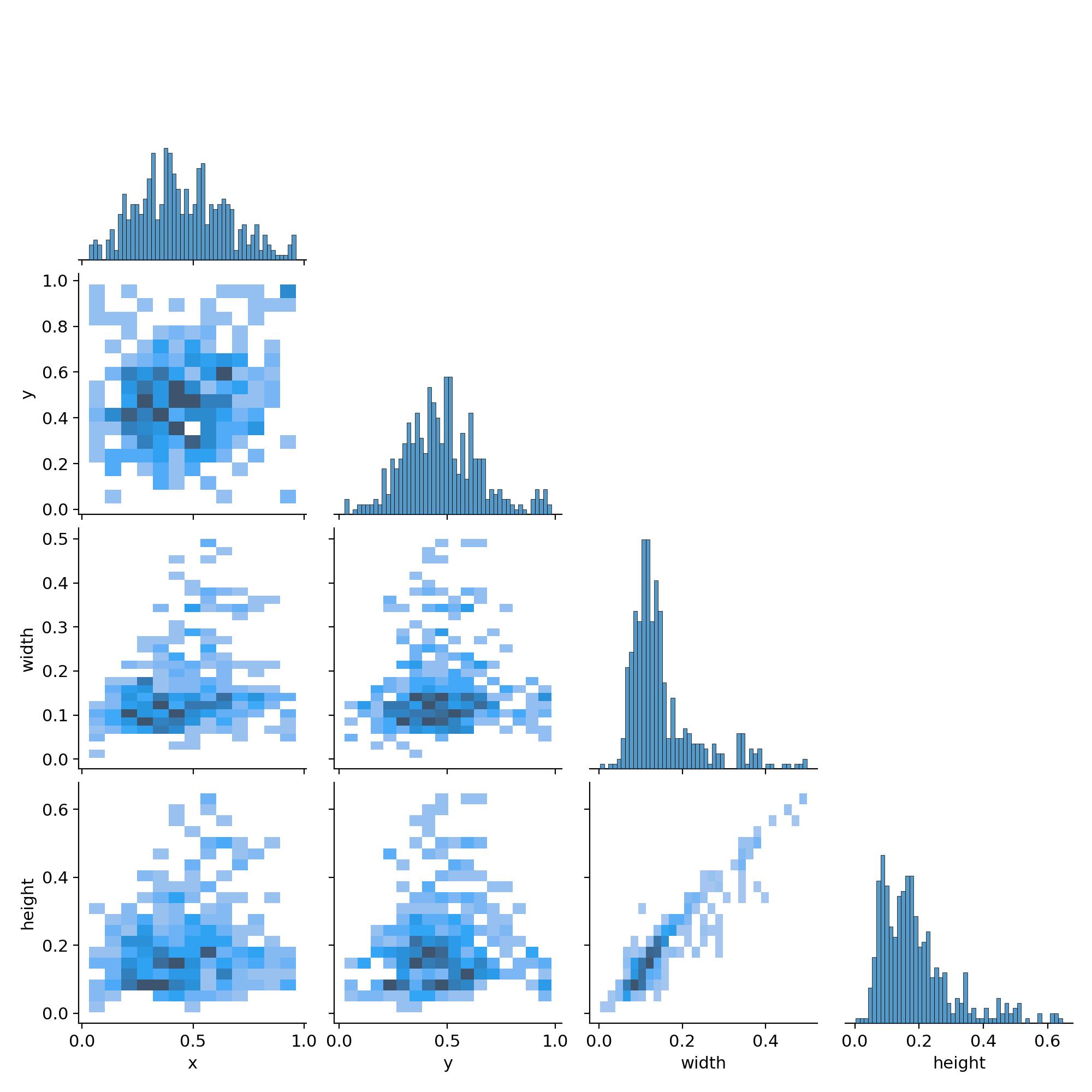

Supplement: Supplemental Information 4 — All of the model parameters can be found in the game.yaml file, the model weights can be found in TrainedModel_V1/weights/best.pt. The validation step has labeled and predicted images in TrainedModel_V1/val_batch0_labels and TrainedModel_V1/val_batch0_pred. [file peerj-cs-10-1826-s004.zip › YoloV8/TrainedModel_V1/labels_correlogram.jpg]

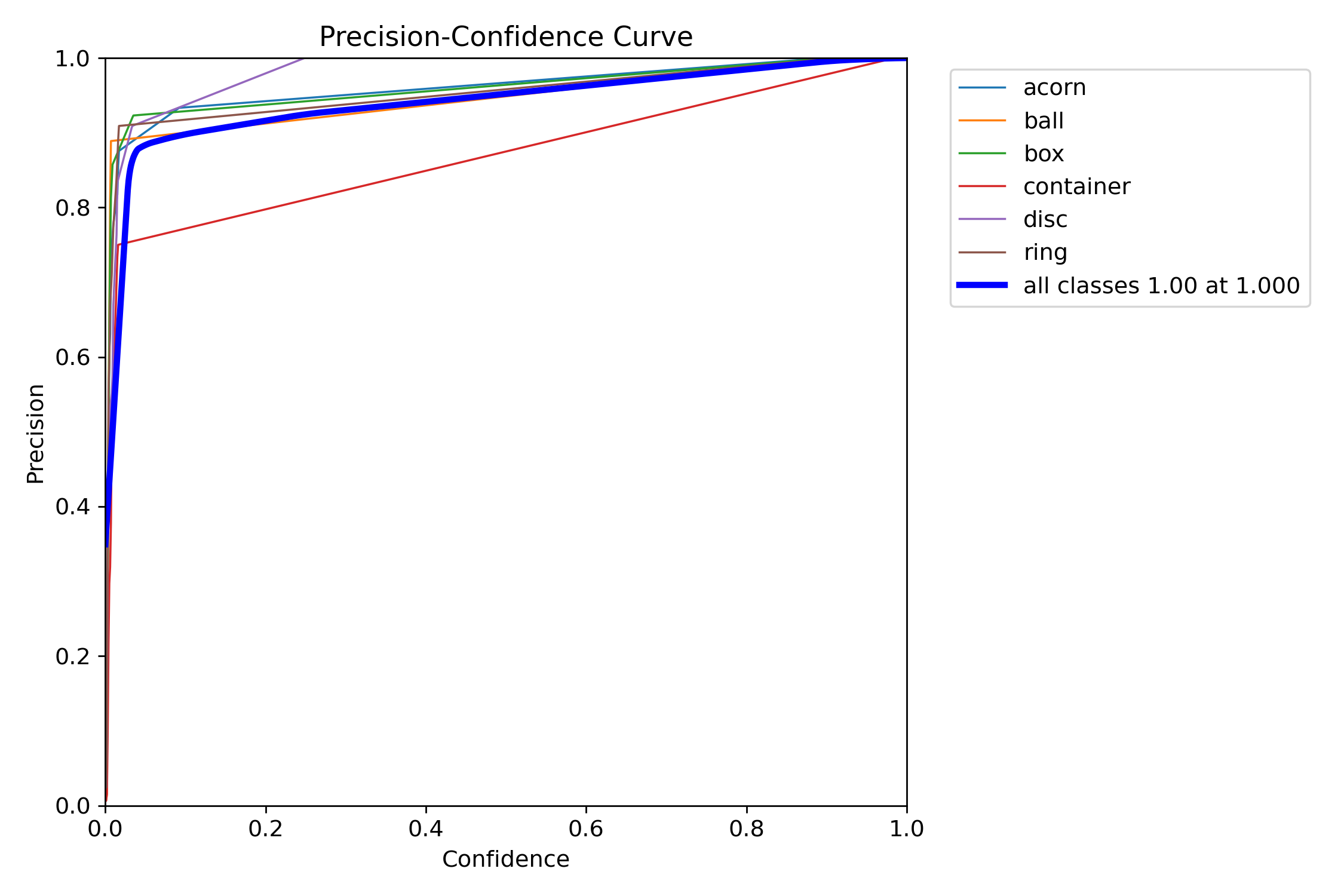

Supplement: Supplemental Information 4 — All of the model parameters can be found in the game.yaml file, the model weights can be found in TrainedModel_V1/weights/best.pt. The validation step has labeled and predicted images in TrainedModel_V1/val_batch0_labels and TrainedModel_V1/val_batch0_pred. [file peerj-cs-10-1826-s004.zip › YoloV8/TrainedModel_V1/P_curve.png]

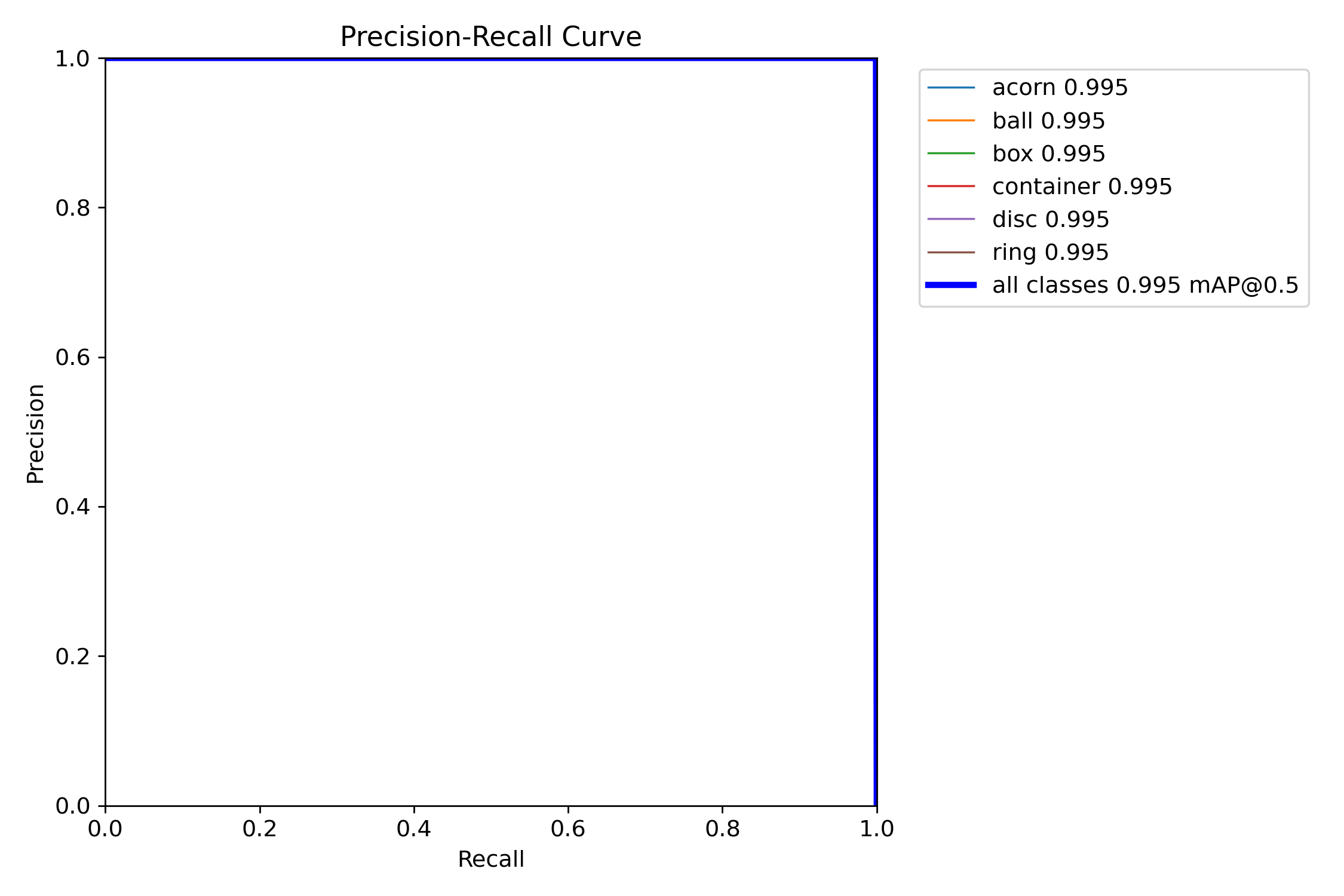

Supplement: Supplemental Information 4 — All of the model parameters can be found in the game.yaml file, the model weights can be found in TrainedModel_V1/weights/best.pt. The validation step has labeled and predicted images in TrainedModel_V1/val_batch0_labels and TrainedModel_V1/val_batch0_pred. [file peerj-cs-10-1826-s004.zip › YoloV8/TrainedModel_V1/PR_curve.png]

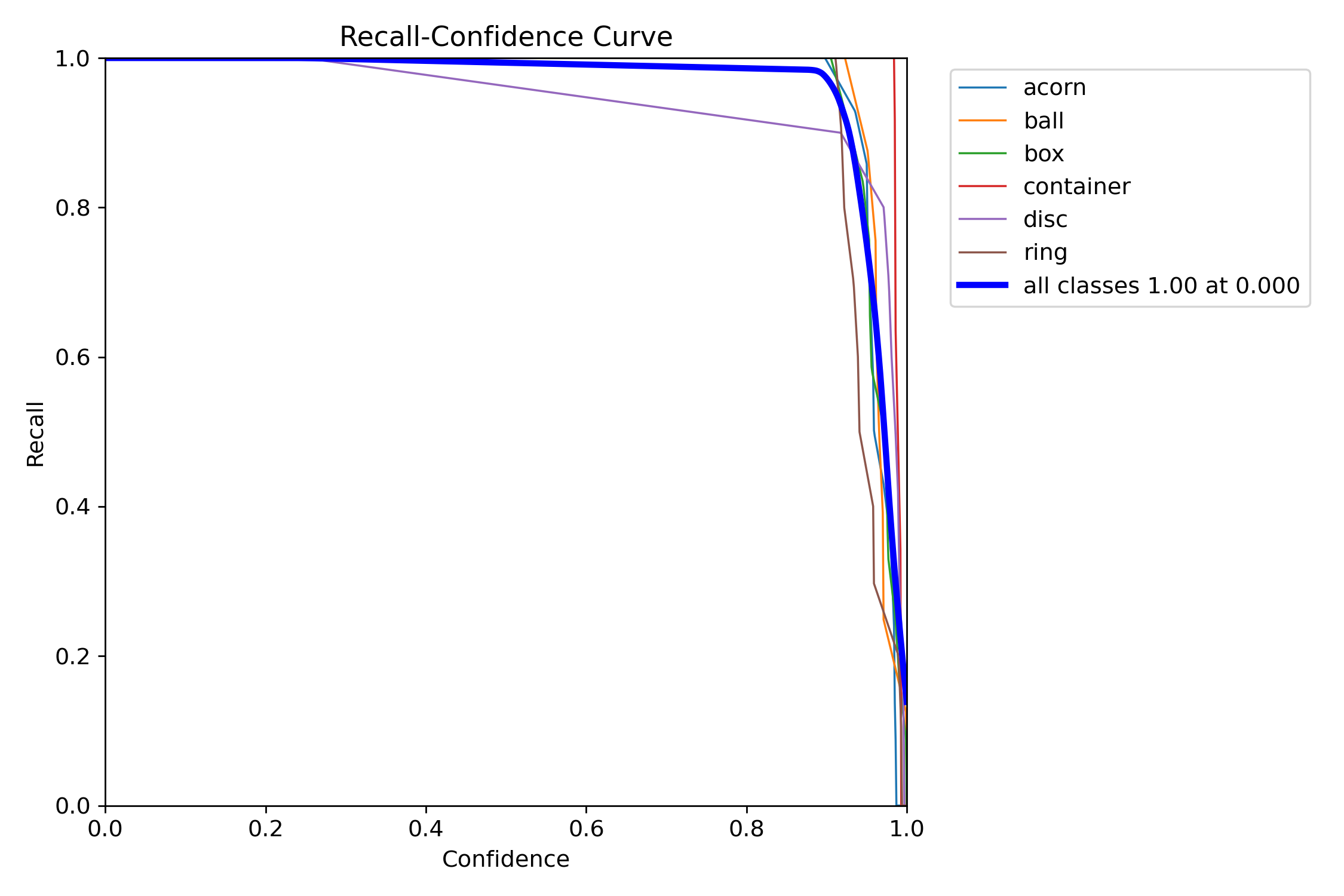

Supplement: Supplemental Information 4 — All of the model parameters can be found in the game.yaml file, the model weights can be found in TrainedModel_V1/weights/best.pt. The validation step has labeled and predicted images in TrainedModel_V1/val_batch0_labels and TrainedModel_V1/val_batch0_pred. [file peerj-cs-10-1826-s004.zip › YoloV8/TrainedModel_V1/R_curve.png]

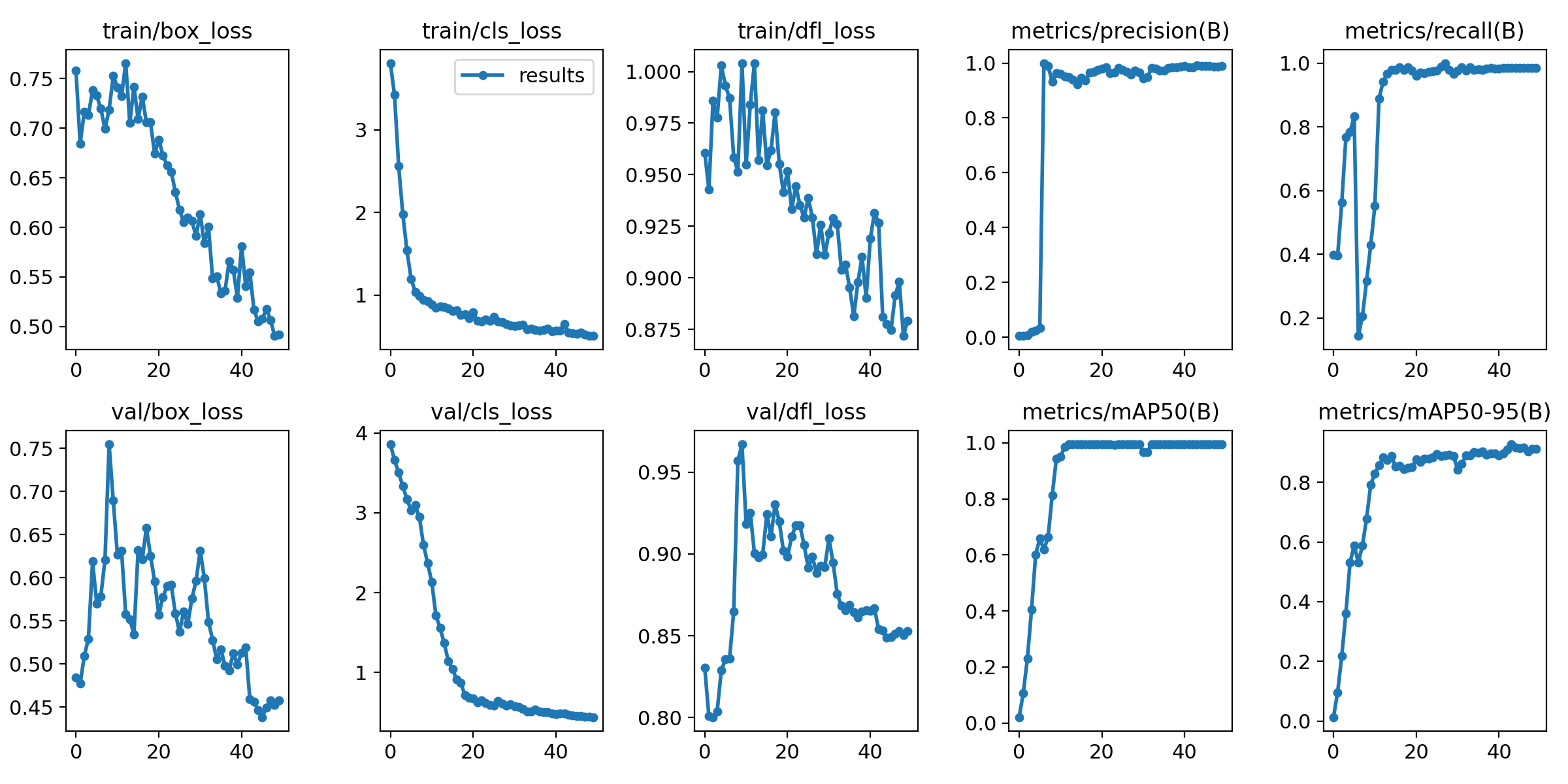

Supplement: Supplemental Information 4 — All of the model parameters can be found in the game.yaml file, the model weights can be found in TrainedModel_V1/weights/best.pt. The validation step has labeled and predicted images in TrainedModel_V1/val_batch0_labels and TrainedModel_V1/val_batch0_pred. [file peerj-cs-10-1826-s004.zip › YoloV8/TrainedModel_V1/results.png]

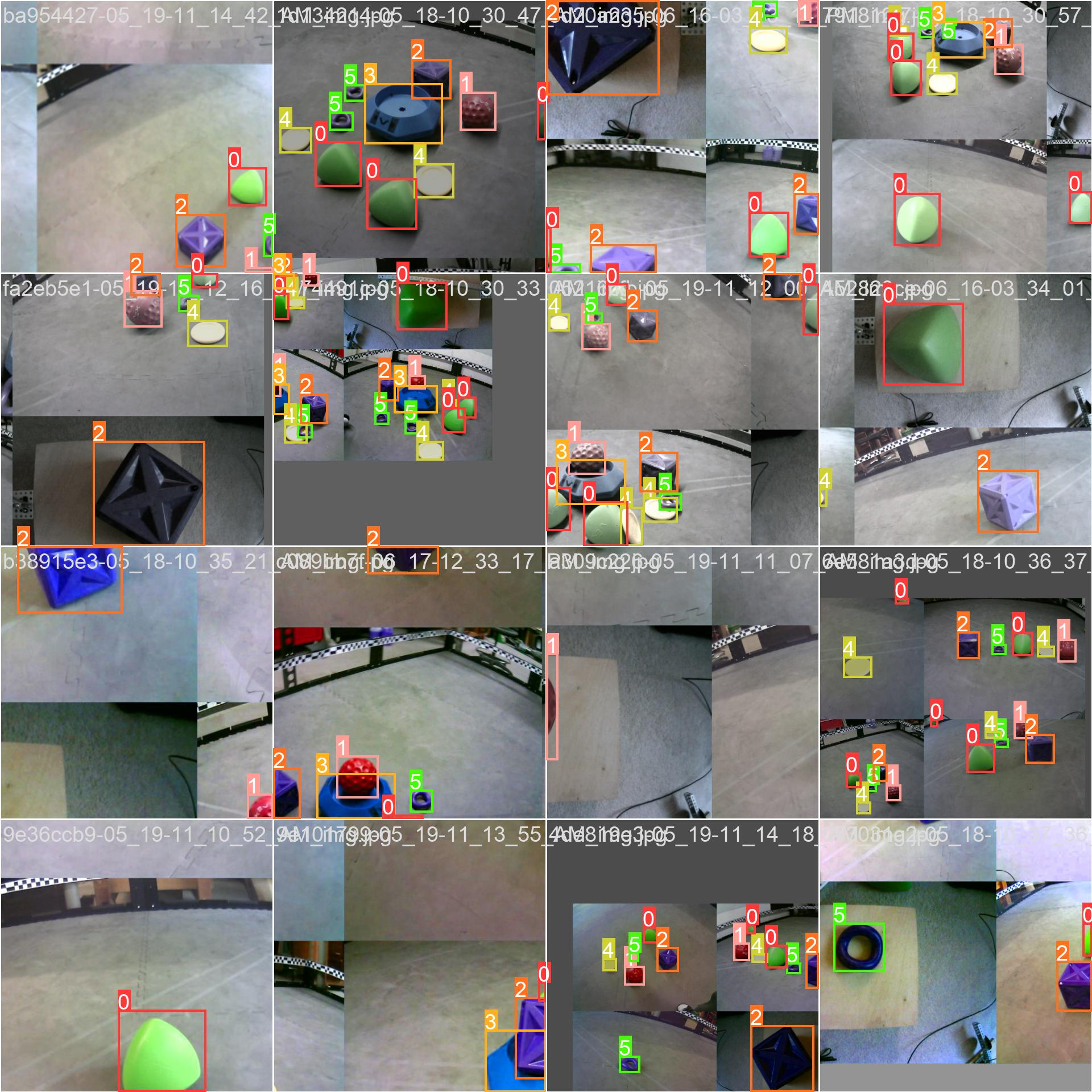

Supplement: Supplemental Information 4 — All of the model parameters can be found in the game.yaml file, the model weights can be found in TrainedModel_V1/weights/best.pt. The validation step has labeled and predicted images in TrainedModel_V1/val_batch0_labels and TrainedModel_V1/val_batch0_pred. [file peerj-cs-10-1826-s004.zip › YoloV8/TrainedModel_V1/train_batch0.jpg]

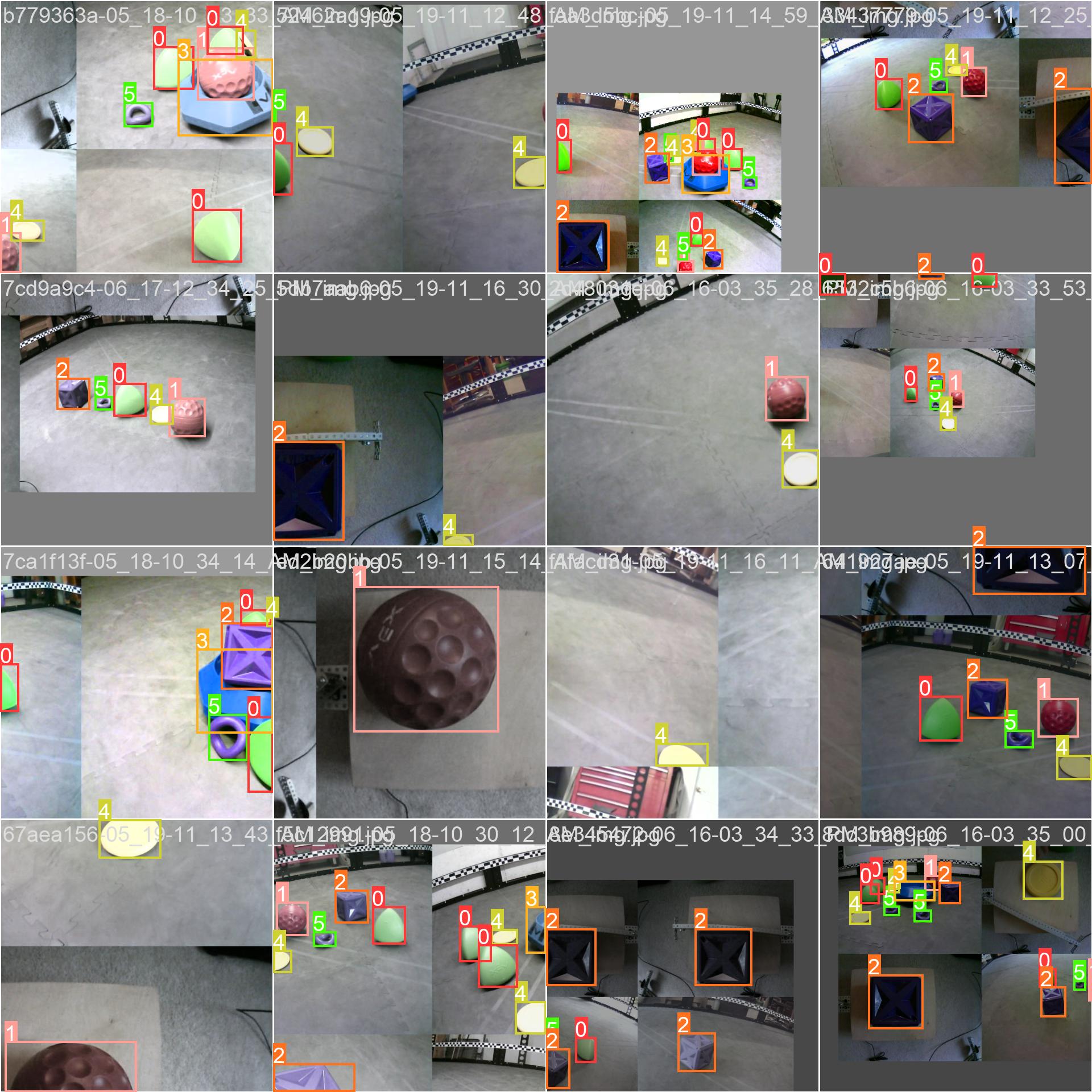

Supplement: Supplemental Information 4 — All of the model parameters can be found in the game.yaml file, the model weights can be found in TrainedModel_V1/weights/best.pt. The validation step has labeled and predicted images in TrainedModel_V1/val_batch0_labels and TrainedModel_V1/val_batch0_pred. [file peerj-cs-10-1826-s004.zip › YoloV8/TrainedModel_V1/train_batch1.jpg]

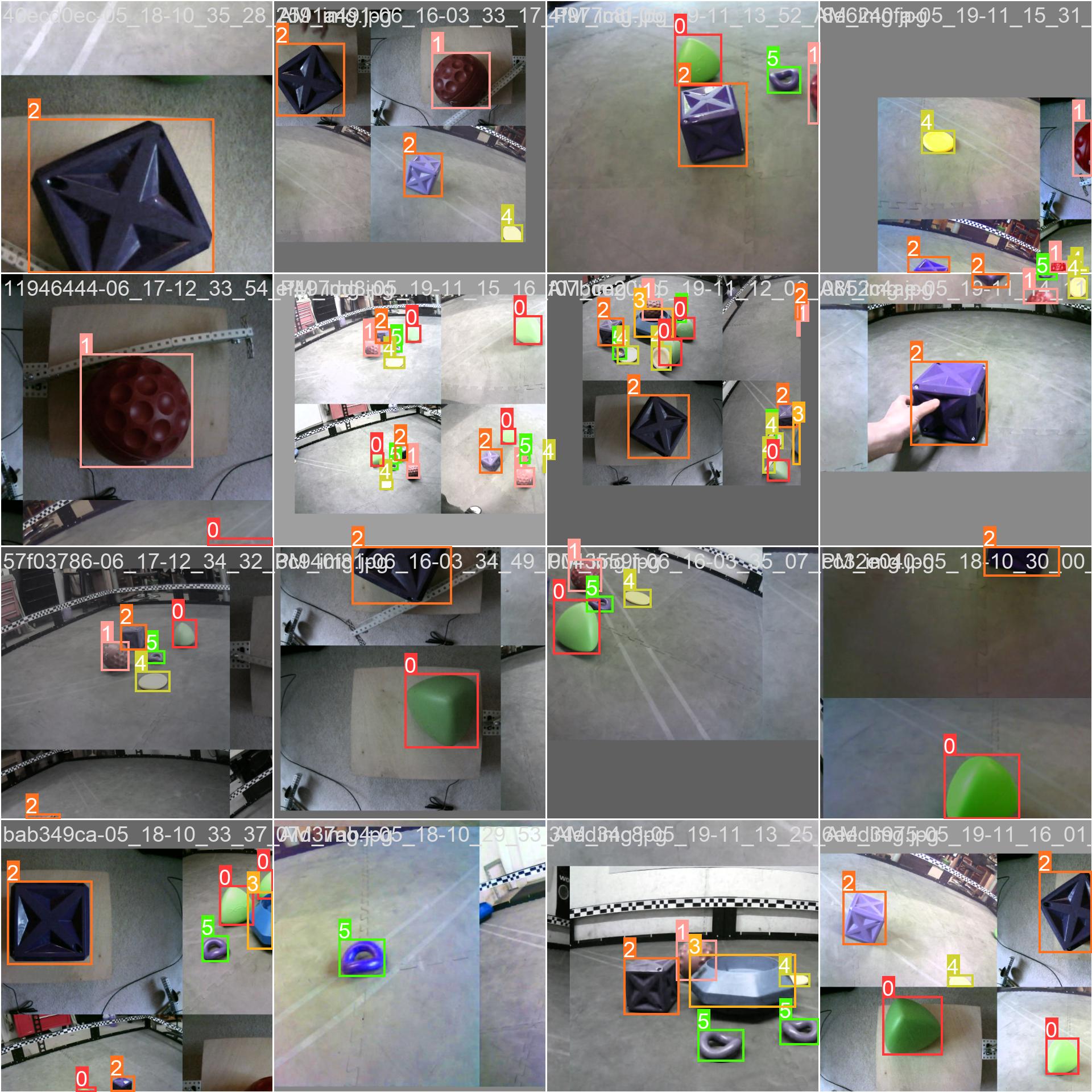

Supplement: Supplemental Information 4 — All of the model parameters can be found in the game.yaml file, the model weights can be found in TrainedModel_V1/weights/best.pt. The validation step has labeled and predicted images in TrainedModel_V1/val_batch0_labels and TrainedModel_V1/val_batch0_pred. [file peerj-cs-10-1826-s004.zip › YoloV8/TrainedModel_V1/train_batch2.jpg]

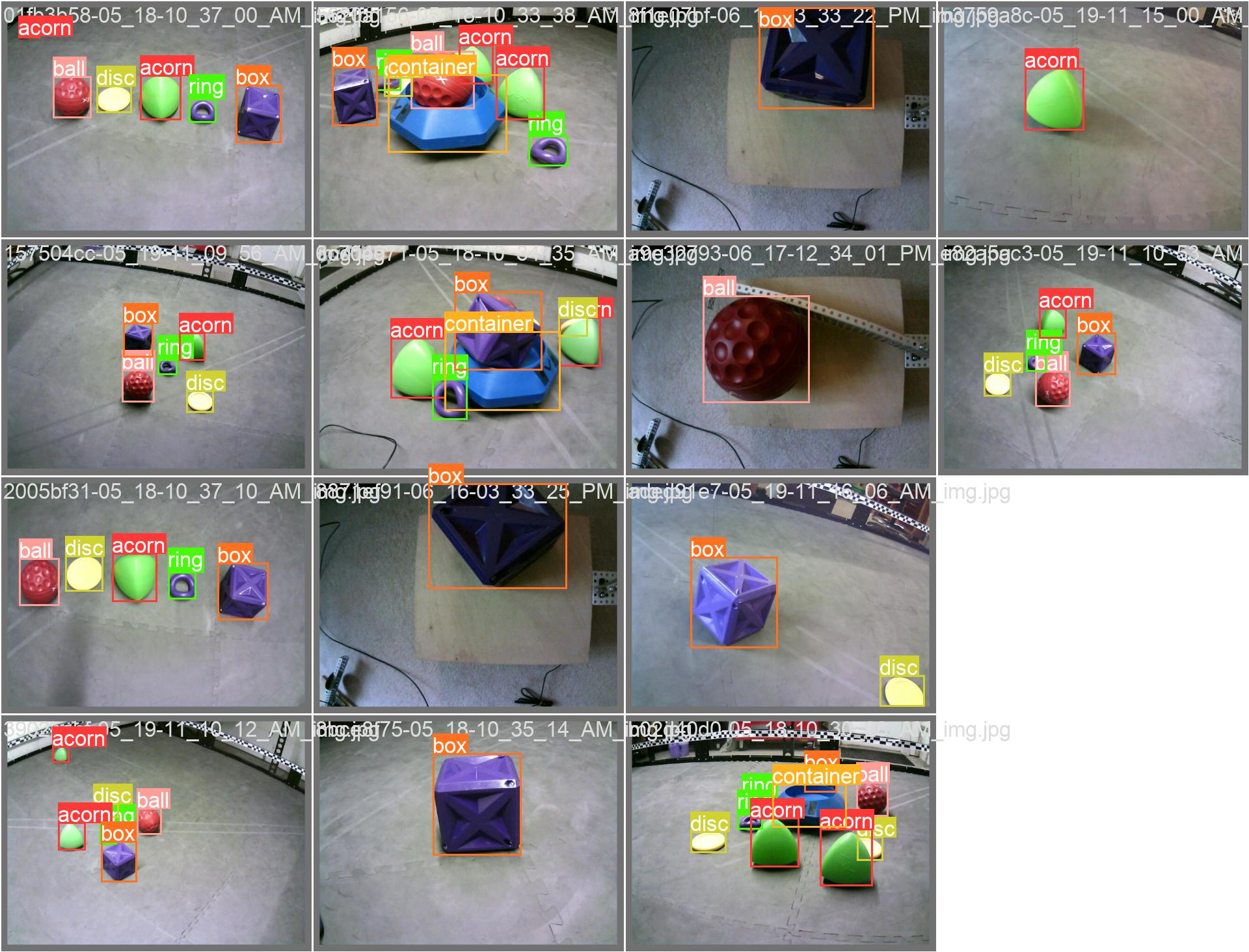

Supplement: Supplemental Information 4 — All of the model parameters can be found in the game.yaml file, the model weights can be found in TrainedModel_V1/weights/best.pt. The validation step has labeled and predicted images in TrainedModel_V1/val_batch0_labels and TrainedModel_V1/val_batch0_pred. [file peerj-cs-10-1826-s004.zip › YoloV8/TrainedModel_V1/val_batch0_labels.jpg]

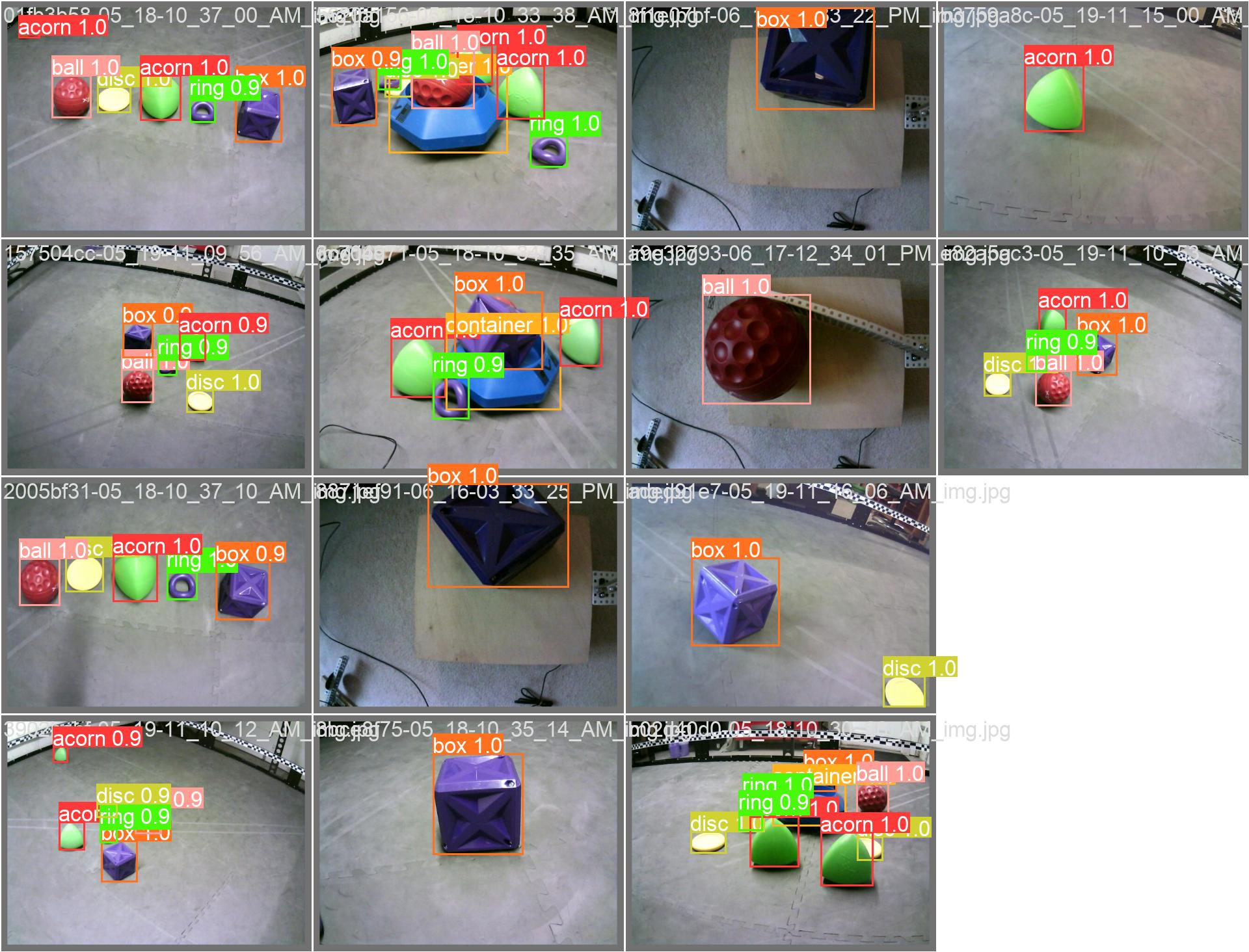

Supplement: Supplemental Information 4 — All of the model parameters can be found in the game.yaml file, the model weights can be found in TrainedModel_V1/weights/best.pt. The validation step has labeled and predicted images in TrainedModel_V1/val_batch0_labels and TrainedModel_V1/val_batch0_pred. [file peerj-cs-10-1826-s004.zip › YoloV8/TrainedModel_V1/val_batch0_pred.jpg]
